# The Antioxidant and Immunomodulatory Potential of *Coccoloba alnifolia* Leaf Extracts

**DOI:** 10.3390/ijms242115885

**Published:** 2023-11-01

**Authors:** Luciana Fentanes Moura de Melo, Jefferson da Silva Barbosa, Maria Lúcia da Silva Cordeiro, Verônica Giuliani de Queiroz Aquino-Martins, Ariana Pereira da Silva, Weslley de Souza Paiva, Elielson Rodrigo Silveira, Déborah Yara A. Cursino dos Santos, Hugo Alexandre Oliveira Rocha, Kátia Castanho Scortecci

**Affiliations:** 1 Laboratory of Plant Transformation and Microscopy Analysis (LPTAM), Cell Biology and Genetics Department, Centro de Biociências, Federal University of Rio Grande do Norte (UFRN), Natal 59078-970, RN, Brazil; lucianafentanes@gmail.com (L.F.M.d.M.); verinhaquino@gmail.com (V.G.d.Q.A.-M.); arianapereirauf@gmail.com (A.P.d.S.); 2 Laboratory of Biotechnology of Natural Polymers (BIOPOL), Biochemistry Department, Centro de Biociências, Federal University of Rio Grande do Norte (UFRN), Natal 59078-970, RN, Brazil; jefferson.barbosa@ifrn.edu.br (J.d.S.B.); wdspaiva@gmail.com (W.d.S.P.); hugo.rocha@ufrn.br (H.A.O.R.); 3 Biochemistry and Molecular Biology Graduation School Programa de Pós-Graduação em Bioquímica, Federal University of Rio Grande do Norte (UFRN), Natal 59012-570, RN, Brazil; 4 Federal Institut of Education, Science and Technology of Rio Grande do Norte (IFRN), São Gonçalo do Amarante 59291-727, RN, Brazil; 5 Northeast Biotecnology Network (RENORBIO), Federal University of Rio Grande do Norte (UFRN), Natal 59078-970, RN, Brazil; 6 Phytochemistry Laboratory, Botany Departament, Bioscience Institut, São Paulo University, São Paulo 05508-070, SP, Brazil; elielson.bio@ib.usp.br (E.R.S.); dyacsan@ib.usp.br (D.Y.A.C.d.S.)

**Keywords:** Cauaçu, leaves, HPLC-DAD, MS/MS mass spectrometry, NO reduction, wound healing, qRT-PCR

## Abstract

Oxidative stress has been associated with different diseases, and different medicinal plants have been used to treat or prevent this condition. The leaf ethanolic extract (EE) and aqueous extract (AE) from *Coccoloba alnifolia* have previously been characterized to have antioxidant potential in vitro and in vivo. In this study, we worked with EE and AE and two partition phases, AF (ethyl acetate) and BF (butanol), from AE extract. These extracts and partition phases did not display cytotoxicity. The EE and AE reduced NO production and ROS in all three concentrations tested. Furthermore, it was observed that EE and AE at 500 μg/mL concentration were able to reduce phagocytic activity by 30 and 50%, respectively. A scratch assay using a fibroblast cell line (NHI/3T3) showed that extracts and fractions induced cell migration with 60% wound recovery within 24 h, especially for BF. It was also observed that AF and BF had antioxidant potential in all the assays evaluated. In addition, copper chelation was observed. This activity was previously not detected in AE. The HPLC-DAD analysis showed the presence of phenolic compounds such as *p*-cumaric acid and vitexin for extracts, while the GNPS annotated the presence of isoorientin, vitexin, kanakugiol, and tryptamine in the BF partition phase. The data presented here demonstrated that the EE, AE, AF, and BF of *C. alnifolia* have potential immunomodulatory effects, antioxidant effects, as well as in vitro wound healing characteristics, which are important for dynamic inflammation process control.

## 1. Introduction

Inflammation is a complex biological response promoted by different sources such as injury, infection, and trauma, and this process produces a burst of reactive oxygen species (ROS) and reactive nitrogen species (RNS) that need to be reduced to avoid cellular damage. This process involves many mediators, cell signaling events, and biochemical cascades. Additionally, to restore and maintain normal physiology, this process is controlled by different mechanisms. It is divided into acute and chronic inflammation [1,2] with many inflammatory markers such as interleukins, interferons, chemokines, tumor necrosis factors, growth factors, stimulating factors, and extracellular matrix (ECM). These markers are subdivided into pro-inflammatory cytokines (IL-1, IL-6, IL-15, IL-17, IL-23, and TNF-α) and anti-inflammatory cytokines (IL-4, IL-10, IL-13, TGF-β, interferon γ (IFNγ)) [3,4,5,6,7]. The inflammation process also involves different cell types and mediators that may regulate cell chemotaxis, migration, and proliferation in a coordinated manner. An imbalance between tissue homeostasis and inflammatory cytokines may have a negative impact on patient health and has been associated with different diseases such as rheumatoid arthritis, inflammatory bowel disease, Crohn’s disease, metabolic disorders, and Alzheimer’s disease [8,9,10,11,12,13].

Medicinal plants have been used for a long time, and they have been a source of various metabolites for the pharmaceutical industry. Many drugs used for the treatment of cancer, cardiovascular diseases, and fever and for other applications have been obtained from medicinal plants or from synthetic molecules derived from these bioactive molecules [14,15]. It has been estimated by the World Health Organization (WHO) that at least 70% of the world’s population uses traditional treatment methods. Furthermore, based on the complex nature of some diseases, such as cancer and inflammatory and degenerative diseases, the identification of medicinal plants has become important as they are considered a reservoir of bioactive molecules. The chemical, biological, and pharmaceutical characterization of these new, as well as old, bioactive molecules and their combination are important for research, innovation, and the pharmaceutical industry [14,16].

The species *Coccoloba uvifera*, *Coccoloba cereifera*, and *Coccoloba mollis* belong to the *Polygonaceae* family and have been characterized for some biological activities, including *Aedes aegypti* larvicidal activity [17], antioxidant activity [18], antifungal activity from root extracts [19], cytotoxic activity and apoptosis induction [20], anti-tyrosinase [21], and antimicrobial activities [22]. Nevertheless, the species *Coccoloba alnifolia* has not been extensively studied. This species, commonly known as Cauaçu, thrives across four distinct Brazilian biomes: the Atlantic and Amazon Rainforests, the Caatinga, and the Cerrado. A previous study [23] observed that six extracts of its leaves were not cytotoxic against NIH/3T3 (murine fibroblast cell line) or tumor cells such as HeLa (human cervical adenocarcinoma cells), SiHa (human cervix squamous cell carcinoma), PC-3 (human prostatic adenocarcinoma cells), B16-F10 (murine melanoma cells), and PANC-1 (human pancreas epithelioid carcinoma cells). In addition, four extracts showed in vitro antioxidant activity in several tests. Building upon these prior data, two extracts (ethanol extract (EE) and aqueous extract (AE)) were selected for assessment as antioxidant agents in the *Caenorhabditis elegans* model. Among these, AE demonstrated the most substantial antioxidant activity. Analyses revealed the presence of gallic acid, *p*-coumaric acid, and vitexin in AE, which are recognized as antioxidant metabolites. These components were identified as the contributors to the antioxidant effects exhibited by AE.

Given the significance and intricacy of the inflammatory process along with its connection to reactive oxygen species (ROS), coupled with the observed antioxidant activity in *Coccoloba alnifolia*, the objective of this study was to assess the potential immunomodulatory effects of the EE, AE, and two partition phases derived from AE (AF and BF). This evaluation aims to shed light on their capability for immunomodulation while also considering the demonstrated antioxidant potential. The data obtained showed that EE and AE extracts were able to reduce ROS (reactive oxygen species), nitric oxide (NO), and phagocytosis. The AF and BF partition phases have antioxidant potential and were able to reduce NO. In addition, these extracts and fractions had a cell migration and wound healing potential, which can be associated with immunomodulatory potential.

## 2. Results

### 2.1. MTT Reduction and Intracellular ROS Production Assay

**Figure 1:**
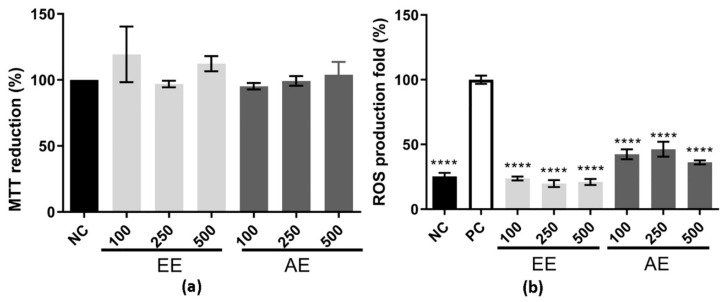
Cytotoxic effect and ROS levels using the RAW 264.7 cell line. EE corresponds to ethanolic extract; AE corresponds to the aqueous extract; and 100, 250, and 500 µg/mL were the three concentrations evaluated. In (**a**), the *Y*-axis corresponds to the MTT reduction percentage, and in the *X*-axis, the different extracts used on the RAW 264.7 cell line. Results were expressed as mean ± standard deviation (SD), and it was performed using one-way ANOVA followed by the Tukey–Kramer test (*p* < 0.05). In (**b**), for the ROS level using the RAW 264.7 cell line, the negative control (NC, black box) was made up of cells grown with only DMEM, while the positive control (PC, white box) was made up of cells incubated with DMEM and LPS at 2 µg/mL, and EE and AE were added to the cell after they had been stimulated with LPS for 24 h. **** indicates a significant difference between the positive control. The *x*-axis corresponds to the different extracts with their concentrations, and the *y*-axis corresponds to the ROS production fold. This experiment was performed in triplicate and repeated three times. Statistical analysis was performed using one-way ANOVA followed by the Tukey–Kramer test (*p* ≤ 0.001).

Melo et al. [17] reported the antioxidant potential of EE and AE both in vitro and in vivo. To gain a better understanding of the diverse biological activities exhibited by these extracts, we investigated their immunomodulatory potential using the RAW 264.7 cell line, along with other activities associated with antioxidant properties. First, the cytotoxicity of the EE and AE was evaluated at different concentrations (100, 250, and 500 µg/mL) using the RAW 264.7 cell line (Figure 1a). It was observed that these two extracts were not cytotoxic by the MTT reduction method. Considering this, the intracellular ROS levels were evaluated using the RAW 264.7 cell line, which was stimulated with LPS as a stress inducer and treated with EE and AE to analyze their effect on ROS levels (Figure 1b). The negative control corresponds to cells that were treated with medium only, whereas the positive control corresponds to cells that were treated with medium plus LPS. Both EE and AE treatment showed a reduction of around ≥50% compared to the PC in all three concentrations tested (100, 250, and 500 µg/mL) (Figure 1b).

### 2.2. EE and AE Immunomodulatory Activity

**Figure 2:**
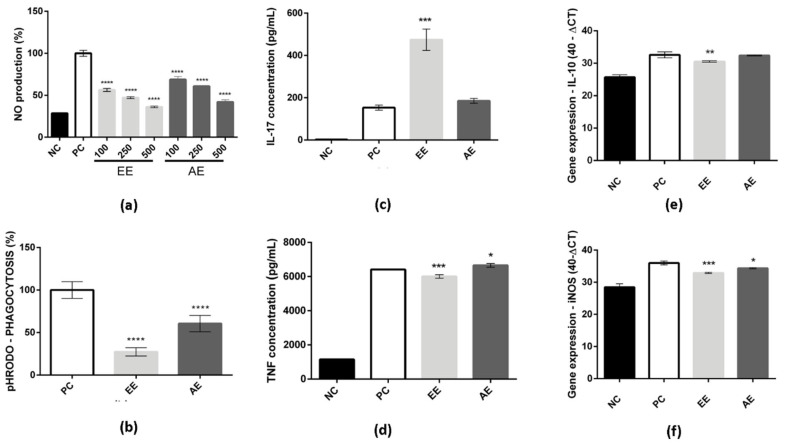
EE and AE effect on RAW 264.7. In (**a**), NO production measurement after LPS and *C. alnifolia* extract treatments. The *x*-axis corresponds to 100, 250, and 500 µg/mL EE or AE concentrations used to treat RAW 264.7 macrophages, and the *y*-axis corresponds to the level of NO production. In (**b**), phagocytosis activity results from RAW 264.7 cells that were treated with EE and AE. The *x*-axis corresponds to different extracts, and the *y*-axis corresponds to pH RODO phagocytosis. **** indicates a significant difference between the positive control and the EE and AE concentrations. The experiments were analyzed using one-way ANOVA followed by the Tukey–Kramer test (*p* ≤ 0.0001). In (**c**,**d**), IL-17A and TNF-α were the cytokines measured after the RAW 264.7 were exposed to LPS and treated with *C. alnifolia* extracts. The *x*-axis corresponds to the different extracts, and the *y*-axis corresponds to cytokine concentration (pg/mL). In (**e**,**f**), mRNA expression was measured by qRT-PCR for the iNOS and IL-10 genes. The *x*-axis corresponds to different extracts, and the *y*-axis corresponds to gene expression. *** (*p* ≤ 0.001), ** (*p* ≤ 0.01), and * (*p* ≤ 0.05) indicate a significant difference between the positive control and the tested concentration (500 µg/mL) of the extracts (EE and EA); statistical analysis was performed using one-way ANOVA followed by the Tukey–Kramer test. Results are expressed as means ± standard deviation (SD). NC, negative control—cells incubated with only DMEM medium; PC, positive control—cells treated with DMEM medium and LPS at 2 µg/mL; EE—ethanolic extract; and AE—aqueous extract. These experiments were performed in triplicate and repeated three times.

In Figure 2a, the effect of dose on NO reduction for both *C. alnifolia* extracts was observed after treatment of RAW 264.7 cells with LPS and subsequent exposure to the plant extracts. Positive control cells (PC) correspond to cells that were treated with LPS and represent 100% of NO production. The negative control (NC) corresponds to cells that were treated with a medium only. When the RAW 264.7 cell line was treated with EE, a reduction of around 50% was observed in NO production at the higher concentration (500 µg/mL). In addition, for AE, a reduction of around 30% in NO production at the 500 µg/mL concentration was also observed. In both extracts, the dose effect was observed according to the extract concentration used (Figure 2a).

The phagocytosis activity was also analyzed, and it was observed that both extracts (EE and AE) were able to reduce the phagocytosis activity of the macrophage RAW 264.7 cell line (Figure 2b). The EE showed a reduction of approximately 30% on phagocytosis, and AE showed a reduction of approximately 50%.

In addition, to understand how these extracts may act in cells, cytokines were measured as they may be directly related to cell signaling and potential mediators of the immune system. The EE stimulated the production of IL-17 when compared to the positive control (Figure 2c) and promoted slight inhibition of TNF-α (Figure 2d). Other cytokines were analyzed, but no differences were observed when compared to the positive control. In contrast, the AE showed similar levels of IL-17 to the positive control (Figure 2c) and slight stimulation of the production of TNF-α when compared to the positive control (Figure 2d). There were no observed differences in other cytokine levels compared to the positive control for AE either. The mRNA expression of iNOS and IL-10 was evaluated. It was observed that both extracts (EE and AE) exhibited a slight inhibition of iNOS expression compared to the positive control (Figure 2e). Furthermore, upon comparing the two extracts, it was found that EE demonstrated superior activity in this regard. Moreover, the inhibition of IL-10 expression, specifically induced by EE, was also observed when compared to the positive control (Figure 2f).

**Figure 3:**
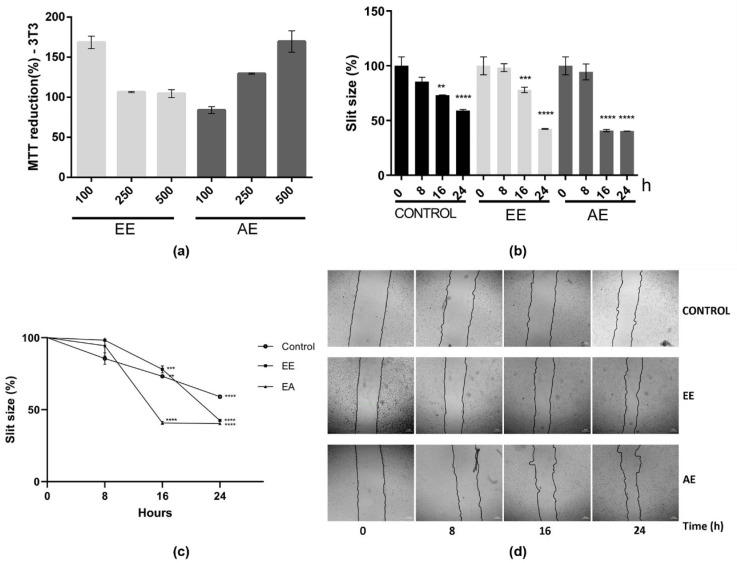
Effects of EE and AE from *C. alnifolia* on cell migration using the 3T3 cell line. (**a**) Cytotoxic effect of EE and AE from *C. alnifolia* in 3T3 cell line. EE, ethanol extract; AE, aqueous extract. The EE and EA were used at three different concentrations: 100, 250, and 500 µg/mL. The *x*-axis corresponds to different extracts, and the *y*-axis corresponds to MTT reduction. (**b**) Graphic using a bar representation of the effect of control, EE, and AE on cell migration. (**c**) Graphic using line representation of the effect of control, EE, and AE on cell migration. The *y*-axis represents the slit size percentage (measurement was determined from the edge of the scratch towards the center, that is, the total distance that the cells moved). *X*-axis corresponds to different treatments (control, EE, or AE) at observation time points (0, 8, 16, and 24 h). (**d**) The images were captured using a Nikon Eclipse inverted microscope with a 40× magnification at 0, 8, 16, and 24 h after scratch and treatment. The scratch area was analyzed using the NIS-Elements AR software ver 5.42.01, and the result was considered as a percentage of scratch closure in relation to the initial area. Results are expressed as mean ± standard deviation (SD). These experiments were performed in triplicate and repeated three times. The control was comprised of cells grown only with DMEM; EE corresponds to 3T3 cells exposed to ethanol extract; and AE corresponds to 3T3 cells exposed to aqueous extract. Statistical analysis was performed using one-way ANOVA followed by the Tukey—Kramer test. **** (*p* ≤ 0.0001), *** (*p* ≤ 0.001), and ** (*p* ≤ 0.01) indicate a significant difference between the control and assay treatment with EE and AE at 500 µg/mL.

Considering that the inflammatory process plays a crucial role in wound healing, the impact of these extracts on cell migration was subsequently examined using the in vitro scratch assay. First, the cytotoxic effects of these extracts on this cell line were evaluated (Figure 3a). Both EE and AE extracts, at different concentrations (100, 250, and 500 µg/mL), did not display any cytotoxic effect. The percentage of cell growth remained consistently at or above 100% (Figure 3a). Accordingly, the concentration of 500 µg/mL was selected for further experiments, considering the non-cytotoxic nature of both extracts at this concentration.

The cell migration assay using the scratch method was assessed at different time points following the initial scratch: 0, 8, 16, and 24 h (Figure 3b–d). Comparing the 16 and 24 h treatments with EE and AE to the control (no extract), a noticeable enhancement in cell migration, leading to the closure of the scratch, was observed (Figure 3b–d). Furthermore, at 24 h of treatment with EE or AE, it was observed that approximately 60% of the scratch had closed, whereas the control group exhibited only 40% closure (Figure 3). This outcome strongly indicates that these two extracts, EE and AE from *C. alnifolia,* promote cell migration and exhibit a notable wound-healing effect.

### 2.3. AE Partition Phases Antioxidant Activity Characterization

Considering that the extract consisted of a mixture of different bioactive molecules and that both EE and AE demonstrated antioxidant and immunomodulatory potential, AE was selected to undergo partitioning into different phases using solvents of increasing polarity (hexane, dichloromethane, ethyl acetate, butanol, and water). These partition phases were analyzed using HPLC-DAD methods. Based on the obtained peaks, only the ethyl acetate (AF) and butanol (BF) phases were selected for further characterization, as depicted in Supplementary Figure S1 and Table 1.

**Figure 4:**
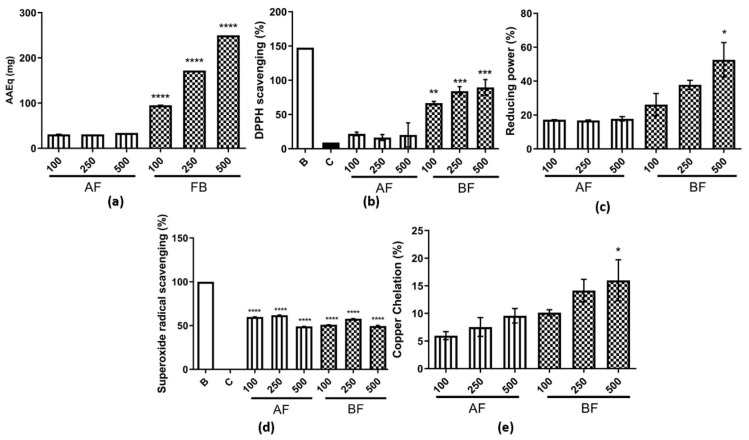
Antioxidant assays for AF and BF from *C. alnifolia*. (**a**) Total antioxidant capacity (TAC). The *x*-axis corresponds to AF and BF partition phases, and the *y*-axis corresponds to the ascorbic acid equivalents in mg (AAEq mg). (**b**) DPPH scavenging radical. The *x*-axis corresponds to AF and BF partition phases used, and the *y*-axis corresponds to the percentage of DPPH scavenging. B corresponds to a control only with DPPH buffer and ethanol 99%, and C corresponds to a control only with DPPH buffer. (**c**) Reducing power. The *x*-axis corresponds to AF and BF partition phases, and the *y*-axis corresponds to the percentage of antioxidant activity. (**d**) Superoxide radical scavenging. The *x*-axis corresponds to AF and BF partition phases, and the *y*-axis corresponds to the percentage of antioxidant activity. B corresponds to a control with reaction solution, and the tube was kept in a dark solution, and C corresponds to a control with reaction solution, and the tube was exposed to a fluorescent lamp. (**e**) Copper chelating. The *x*-axis corresponds to AF and BF partition phases, and the *y*-axis corresponds to the percentage of copper chelation. For all assays completed, the AF and BF partition phases were used in three concentrations: 100, 250, and 500 μg/mL. Each assay was conducted in triplicate and repeated three times. W and C correspond to the control tubes with only reagents without samples; W’s tube was kept in the dark, and C’s tube was exposed to light. The results were analyzed using an ANOVA and Tukey´s test. * corresponds to a significant difference between fractions at *p* < 0.01, ** corresponds to a significant difference between the extracts at *p* < 0.05, *** corresponds to a significant difference between the extracts at *p* < 0.01, and **** corresponds to a significant difference between fractions at *p* < 0.001.

The first in vitro antioxidant activity analyzed was the total antioxidant capacity—TAC (Figure 4a). It was observed that both AF and BF exhibited activity, but it was higher for BF. The assay used in this study measures the sample’s ability to donate electrons, thereby reducing reactive species. Furthermore, a dose-dependent effect was observed for BF, with higher activity observed compared to AF (Figure 4a). In addition to the aforementioned assay, the DPPH was also conducted to evaluate antioxidant activity; as a result, this activity was also observed for both analyzed partition phases. However, the BF exhibited the highest activity among them (Figure 4b). The reducing power assay showed an activity of 20% for AF, and it was approximately 60% for BF at 500 µg/mL (Figure 4c). For the superoxide radical scavenging assay, activity was observed for both partition phases in a similar manner, higher than 50% when compared to control (Figure 4d). An important activity observed in these two partition phases was the copper chelating activity (Figure 4e), which was not observed previously for AE [23]. In addition, for all these antioxidant assays assessed, BF was found to have the highest antioxidant activity.

### 2.4. C. alnifolia Partition Phases Immunomodulatory Activity

**Figure 5:**
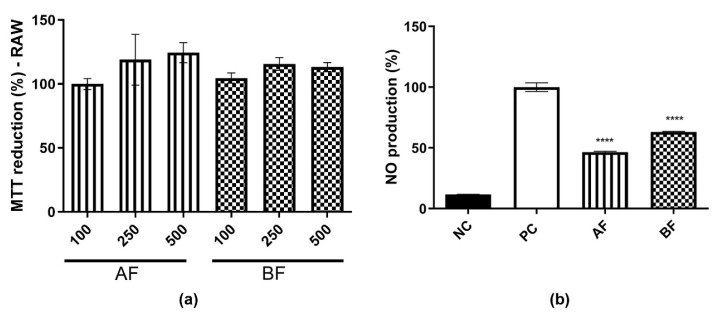
AF and BF effect on NO production. (**a**) MTT reduction on RAW 264.7 cell lines treated with AF and BF partition phases at 100, 250 e, and 500 µg/mL. The results were expressed as average ± SD. It was repeated three times, and it was carried out in triplicate. In (**b**), NO production measurement after LPS and AF and BF partition phases from *C. alnifolia* treatment at 500 µg/mL concentration. The *x*-axis corresponds to the NC treatment with medium only, the positive control PC corresponds to the cell line treated with LPS at 2 µg/mL, and the value obtained corresponds to 100% AF and BF partition phases from *C. alnifolia* treatment at 500 µg/mL. *Y*-axis corresponds to the level of NO production. The results were analyzed using an ANOVA and Tukey´s test. **** corresponds to a significant difference between partition phases at *p* < 0.001.

Based on the antioxidant potential of AF and BF, it was important to evaluate whether these two partition phases were also able to reduce NO, as observed previously for AE. First, it was observed that these two partition phases do not have any cytotoxicity effect on this cell line (Figure 5a). Subsequently, the evaluation of NO production at a concentration of 500 µg/mL was conducted. It was observed that both the AF and BF partition phases were effective in reducing NO production (Figure 5b). Furthermore, when compared to the AE extract (Figure 2a), these partition phases demonstrated a superior ability to reduce NO levels, achieving reductions of approximately 40–50%.

**Figure 6:**
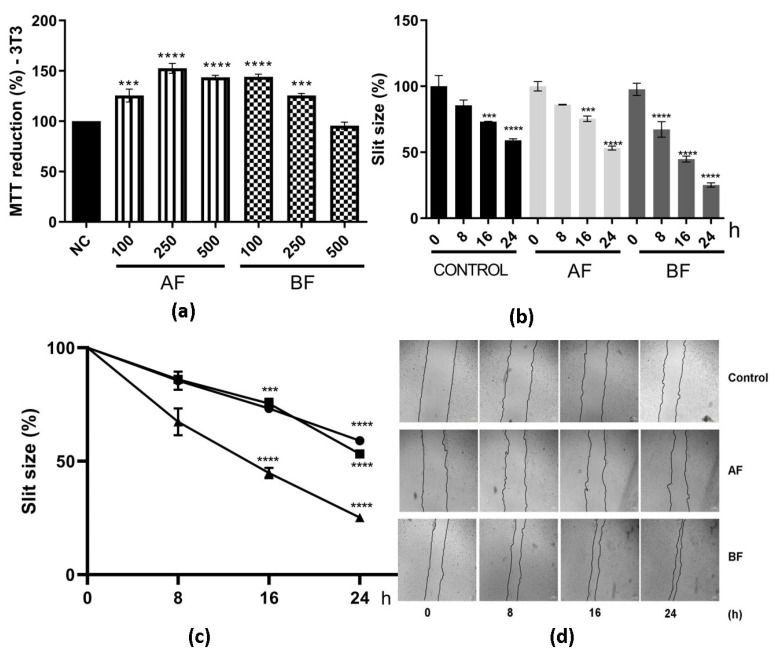
Effects of AF and BF from *C. alnifolia* on cell migration. (**a**) MTT reduction. The *x*-axis corresponds to AF and BF fractions at 100, 250, and 500 µg/mL, and the *y*-axis corresponds to MTT reduction. (**b**) Graphic using a bar representation of the effect of control, AF, and BF on cell migration. (**c**) Graphic using line representation of the effect of control, AF, and BF on cell migration. *Y*-axis represents the slit size percentage (measurement was determined from the edge of the scratch towards the center; that is, the total distance that the cells moved). *x*-axis corresponds to different treatments (control, AF, or BF) at observation time points (0, 8, 16, and 24 h). (**d**) The images were captured using a Nikon Eclipse inverted microscope with a 40× magnification at 0, 8, 16, and 24 h after scratch and treatment. Results are expressed as mean ± standard deviation (SD). The scratch area was analyzed using the NIS-Elements AR software ver5.42.01, and the result was considered as a percentage of scratch closure in relation to the initial area. These experiments were performed in triplicate and repeated three times. The control was cells grown with only DMEM; AF corresponds to 3T3 cells exposed to ethyl acetate partition phases, and BF corresponds to 3T3 cells exposed to butanol partition fraction. Statistical analysis was performed using one-way ANOVA followed by the Tukey—Kramer test. **** (*p* ≤ 0.0001), and *** (*p* ≤ 0.001) indicate a significant difference between the control and assay treatment with AF and BF at 500 µg/mL.

The potential of these two partition phases on cell migration by the scratch method was also evaluated (Figure 6). First, it was observed that there was no cytotoxicity effect on these cell lines at different concentrations (Figure 6a). Then, cell migration was assessed, and it can be observed that BF had 75% on cell migration and AF had 50% at 24 h (Figure 6b–d). The percentage of cell migration was higher for these two partition phases when compared to extract AE and the control (Figure 3 and Figure 6).

### 2.5. C. alnifolia Extracts and Partition Phase Characterization Using Zebrafish Model

**Figure 7:**
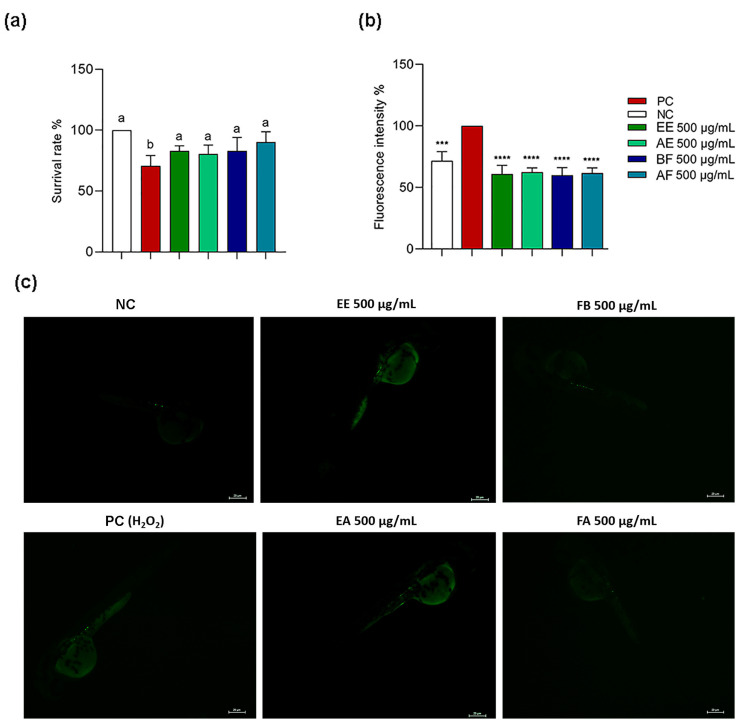
Protective effect of *C. alnifolia* extracts on ROS production after exposure to hydrogen peroxide (H_2_O_2_) in zebrafish embryos. (**a**) corresponds to survival rate from the zebrafish embryos after treatment. (**b**) Graph representation of fluorescent rate from the embryos after treatment. (**c**) Fluorescence from the embryos after treatment with 24 h treatment with DCFH-DA. EE (ethanolic extract), EA (aqueous extract), FB (butanol partition fraction), and FA (acetate partition fraction). NC, negative control—nursery water only; PC, positive control, which contains nursery water and H_2_O_2_. In (**a**) Different letters (a, b) correspond to statistical significance between groups (*p* < 0.05). In (b) *** correspond to statistical significance between groups (*p* < 0.05). **** (*p* ≤ 0.0001).

The in vitro data showed an antioxidant and anti-inflammatory potential for *C. alnifolia* samples. Then, it was observed that these samples did not have an embryotoxicity; then, whether these extracts and partition phase could have a ROS protective effect when zebrafish embryos were treated with stressor agents such as H_2_O_2_ and *C. alnifolia* samples was evaluated (Figure 7). It was observed that the EE, AE, BF, and AF were able to protect these animals against the presence of H_2_O_2,_ as observed by the survival rate (Figure 7a) and by fluorescence intensity (Figure 7b,c). For both conditions, it was observed the sample values were similar to the negative control. These data reinforce the previous results obtained using cell lines.

### 2.6. BF Partition Phase Characterization

**Figure 8:**
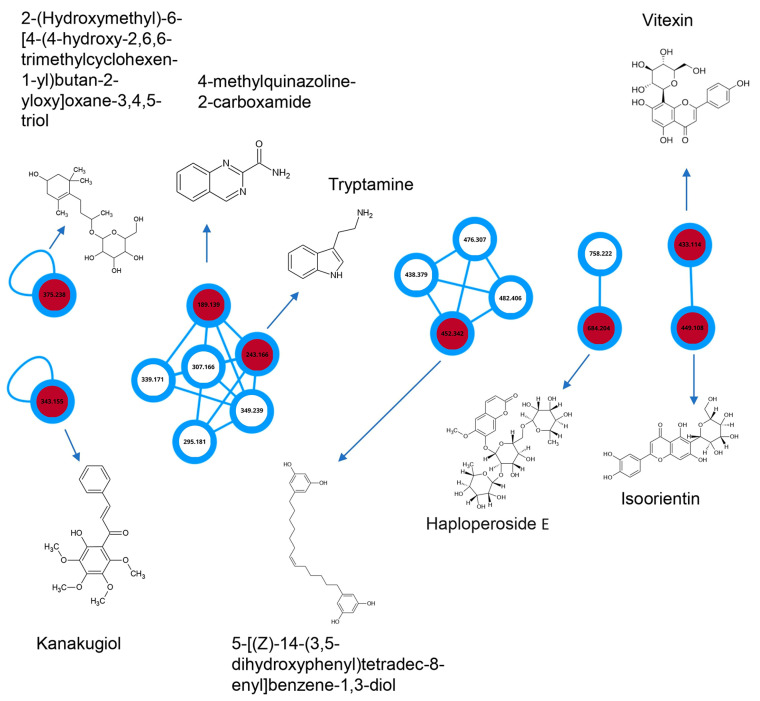
Molecular network from BF partition phase. In the network, circles are used to represent different compounds. Unfilled circles indicate minority compounds, while the red-filled circles represent major compounds. The number displayed inside each circle corresponds to the molecular mass of the respective compound.

Considering the observed potential of the BF partition phase from *C. alnifolia* in promoting cell migration, wound healing, and exhibiting in vitro and in vivo antioxidant activity, this sample was subjected to analysis by MS/MS mass spectrometry (Table 2). A molecular network was constructed with the assistance of GNPS, highlighting the interactions between the compounds. The annotated molecules are described in Table 2. In Figure 8, it can be observed that 4-methylquinazoline-2-carboxamide interacts with tryptamine, while the other molecule interacts with vitexin and isoorientin.

The interaction of the annotated compounds from Table 2 can be seen in Figure 8.

## 3. Discussion

It has been observed that many chronic diseases, such as atherosclerosis, Alzheimer’s disease, type 2 diabetes mellitus, cardiovascular disease, and cancer, may be associated with the redox imbalance from ROS/RNS, which may impact cells and tissues by multiple mechanisms [24,25,26]. Excessive ROS/RNS production may initiate an inflammatory process that is a dynamic response of the cells and tissues against different agents such as microorganisms, antigens entrance, chemicals, obesity, or any cell/tissue damage [2,27]. This process involves the interaction of many mediators (pro- and anti-inflammatory), signals, and cell types. This response needs to be controlled to restore normal physiology and to avoid any other disorder [2,24,27].

Drugs to control the inflammatory process have many side effects; for this reason, the pharmaceutical industry has been searching for new biomolecules from medicinal plants that can be used for these treatments with fewer negative effects. [2,28]. The reduction of oxidative stress and inflammatory effects was found to be an excellent effect of the use of polyphenol and flavonoid compounds [24,29,30]. Molecular pharmacology has been attempting to identify targets/pathways and possible crosstalk from these biomolecules present in medicinal plant extracts to correlate their action with ROS/RNS and inflammation [2,31,32,33,34].

Numerous efforts have been made to identify the biomolecules from medicinal plants and to correlate them with their biological and pharmacological activities [35]. Melo et al. [23], working with six *C. alnifolia* leaf extracts, observed an important antioxidant activity using in vitro and in vivo assays. In addition, TLC showed the presence of gallic acid, *p*-coumaric acid, and vitexin. These molecules were known to have antioxidant activity and anti-inflammatory roles [24,29,36,37]. Here, the potential immunomodulatory effects of the extracts, EE, AE, and two partition phases, AF and BF, were investigated. The idea of making partition phases was based on the extracts’ characteristics, which are a mixture of different biomolecules; additionally, such extracts can have synergistic or antagonistic effects [38,39]. The pharmaceutical industry often prefers to work with a pure molecule, but this does not guarantee that such an isolated molecule has the same activity and efficiency as the extract/fraction from which it originated [39]. Therefore, the AE (aqueous extract), the form used in traditional medicine, was fractionated, and the FA and BF were chosen by the HPLC-DAD profile obtained. The MS/MS spectrometric analysis from BF showed the presence of isoorientin, vitexin, kanakugiol, tryptamine, and other molecules in small proportion. These biomolecules are known to have antioxidant activity and anti-inflammatory roles [24,29,30,36,37]. In addition to that, Yang et al. [40] have reported that Haploperoside exhibits the potential to inhibit the production of NO induced by LPS at a concentration of 40 μM in RAW 264.7 macrophages. Hence, the presence of Haploperoside E in this study suggests that it may also contribute to the biological potential of *C. alnifolia*.

Furthermore, it is important to know the potential activities of these biomolecules to better understand the biological activities identified here. Exemplifying this, it can be noted that an important characteristic observed for all extracts and partition phases analyzed in this work was the absence of cytotoxic effects on cells. This is relevant if, in the future, such extracts are planned to be used in the human diet, for example. These data are in agreement with the effects observed before in other plants or pure molecules, specifically that *p*-coumaric, gallic acid, and isovitexin show no cytotoxic effect on A549 [41] and RAW 264.7 cells [30,37,42,43,44].

The inflammatory response is a complex process with different receptors, mediators, cell signaling, and crosstalk pathways [2,24,27]. In this process, the excess of ROS/RNS can be an inflammatory stimulus promoting the synthesis and secretion of pro-inflammatory cytokines, as well as activating macrophages that trigger inflammatory responses and produce NO, which is an important inflammatory mediator and is released in the cellular response to this process [24,36,45]. The data presented here using macrophage cell line RAW 264.7 in the presence of LPS showed that EE and AE were able to promote the reduction of ROS, and these extracts had a phagocytosis activity. In addition, these extracts and the partition phases AF and BF were able to reduce NO production. Asgharpour et al. [34], working with ethanol extract from propolis (PEEP), rich in phenolic compounds, observed the activity to reduce ROS and NO. Furthermore, different species from Polygonaceae had their anti-inflammatory activity characterized. For example, the *Polygonum odoratum* Lour (extracts from leaf and stem) is rich in phenolic compounds [46], *Fagopyrum* spp. is rich in flavonoids, such as orientin, vitexin, and their isomers [47], *Persicaria chinensis* is rich in flavonoids and phenolic acid (caffeic acid, kaempferol, quercetin) [48], and *Polygonum hydropiper* L. is rich in quercetin [4]. Vitexin and *p*-coumaric acid can reduce the levels of ROS and RNS in Neuro-2a cells stimulated with aggregates of beta amyloid peptides [49] capable of reducing ROS production in A549 cells stimulated with LPS [41]. Treatment of *C. elegans* with *p*-coumaric acid resulted in a significant reduction in intercellular ROS levels, which suggests the in vivo antioxidant capacity of *p*-coumaric acid and vitexin [18]. These data reinforce our results for *C. alnifolia* extracts and fractions and propose that these biomolecules can be a characteristic of this family, but the proportion and combination of biomolecules may be different.

During the inflammatory process, different mediators may be induced as cytokines that are divided into pro-inflammatory cytokines and anti-inflammatory. The pro-inflammatory cytokines are represented by IL-1, IL-6, IL-15, IL-17, IL23, and TNFα, and anti-inflammatory cytokines formed by IL-4, IL-10, IL-13, TGF-β, and IFNγ [15,31]. TNF-α is considered an important mediator with different activities in several cell types. Cytokines may have different roles in this complex process through the activation of intracellular signaling pathways, regulating protein synthesis, migration, proliferation, and cell differentiation. These mediators may be acting antagonistically, additively, or synergistically in the same biological process [49,50]. Phenolic compounds and flavonoids may participate in the inhibition of inflammatory mediators, such as iNOS, cyclooxygenase (COX-2), and the cytokines IL-1β, TNF-α, and IL-10 [49,51]. Suppressed NF-κB may regulate several pro-inflammatory mediators, including pro-inflammatory cytokines and transcription factors [52,53,54,55]. PEEP decreased the expression of COX-2, IL-1β, and IL-6 genes [34]. The results presented here showed that EE and AE reduced NO and may have an immunomodulatory activity as EE modified the IL-17 expression.

Moreover, wound healing is also associated with the inflammation process and ROS. It can be divided into three phases: The first phase is associated with tissue damage that may induce ROS/RNS production and, consequently, an inflammation process as a defense mechanism from tissue. The second phase involves proliferation by cell migration, collagen synthesis, and extracellular matrix formation. The third phase is characterized by tissue remodeling [56,57]. It was observed that the *C. alnifolia* extracts and fractions promoted cell migration of approximately 60% in 24 h. These results were in agreement with the presence of IL-17, a pro-inflammatory cytokine important for tissue repair [32,58,59,60]. Extracts and fractions enriched with flavonoids showed a potential of 50% migration in 24 h and 90% or more after 48 h of treatment [10]. *P. vera* bark fractions were analyzed for cell migration, and it was observed that the dichloromethane fraction displayed better activity, closing the wound by 50.63% in 48 h [12]. These data corroborate ours since the presence of vitexin, isoorientin, kanakugiol, *p*-coumaric, and other molecules were identified in *C. alnifolia* extracts and fractions. These data reinforce that the Polygonaceae family, especially *C. alnifolia*, produces biomolecules that have antioxidant and immunomodulatory effects through NO/ROS reduction and phagocytosis. These data were supported by in vivo assays using the zebrafish model. Chahardehi et al. [61] have shown that the use of embryos and larvae was a suitable model for screening pharmacologic activities such as toxicity and antioxidants. Here, the data presented with *C. alnifolia* samples provided support to biochemical and cell assays.

Identifying and understanding how these biomolecules act in this dynamic antioxidant and inflammatory process and how this interaction network works will provide the possible mechanism of action that is important for nutraceutical and drug innovation in different diseases such as cardiovascular disease [62], liver disease [63], and cancer [64] and others. Tasneem et al. [2] made a schematic representation of the important pathways related to the inflammation process and included possible plant extracts and isolated compounds. This highlights the importance of this work in providing, through the extracts analyzed here, a possible safe, natural, and efficient alternative for drug development. It also sheds more light on the mechanisms of action of biomolecules from plants that have long been used in folk culture as a medicinal source.

## 4. Materials and Methods

### 4.1. Materials

Cell lines used in this study were NIH/3T3 (ATCC CRL-1658), 3T3 (ATCC CCL-164), and RAW 264.7 (ATCC TIB-71) (Rockville, MD, USA). The chemical reagents 3-(4,5-dimethylthiazolyl-2)-2,5-diphenyltetrazolium bromide (MTT), Griess reagent, and 2′,7′-dichlorofluorescin diacetate (DCFH-DA) were purchased from Sigma Chemical Company, (St. Louis, MO, USA). Dulbecco’s modified Eagle’s medium (DMEM) and fetal bovine serum (FBS) were obtained from CULTILAB (Campinas, SP, Brazil). Penicillin and streptomycin were obtained from Gibco (Fort Worth, TX, USA). SYBR Green Master Mix and High-Capacity cDNA Reverse Transcription Kit were purchased from Applied Biosystems (Foster City, CA, USA). The ReliaPrep RNA Cell Miniprep System was purchased from Promega (Madison, WI, USA). DNAse was obtained from Ambion (Life Technologies, Carlsbad, CA, USA). pHrodo™ Red and Green BioParticles ^®^ Conjugate for the Phagocytosis kit was purchased from Invitrogen™ (USA & Canadian). The cytokine analysis kit was purchased from BD Biosciences (San Jose, CA, USA). Lipopolysaccharide (LPS, Escherichia coli 055: B5) was purchased from Santa Cruz Biotechnology (Dallas, TX, USA). All other solvents and chemical products were of analytical grade and commercially available.

### 4.2. Biological Material

*Coccoloba alnifolia* leaves were harvested from Parque das Dunas in the State of Rio Grande do Norte, Brazil, with geographic coordinates in UTM zone 250,257,315 m E 9,357,108 m N (GPS Garmin Etrex, Olathe, KS, USA). The harvest was conducted as previously described [23]. The material had the number 54064-1 SISBIO and a voucher number UFRN17133 from the UFRN herbarium [23].

### 4.3. Extraction

The ethanolic extract (EE) and aqueous extract (AE) were prepared as previously described [23].

### 4.4. Lineage and Cell Culture

The murine macrophage cell line RAW 264.7 (ATCC number TIB-71) and the NIH/3T3 cell line (ATCC CRL-1658) were cultured in DMEM culture medium supplemented with fetal bovine serum (FBS) (10% *v*/*v*) and antibiotics (100 U/mL penicillin and 100 μg/mL streptomycin) and kept in a humidified 5% CO_2_ atmosphere at 37 °C. To maintain the culture, the culture medium was changed every three days, and when the cells reached 80% confluency, the cells were sub-cultured.

### 4.5. MTT Reduction Assay

RAW 264.7 macrophages and NIH/3T3 cell lines were grown in a culture flask using Dulbecco’s Modified Eagle Medium (DMEM) supplemented with FBS (10% *v*/*v*) and antibiotics (100 U/mL penicillin and 100 μg/mL streptomycin) and maintained in a humidified 5% CO_2_ atmosphere at 37 °C. To analyze the effect of EE and EA on cell viability by MTT reduction [65], the two cell lines were grown in 96-well plates until they reached a density of 1 × 10^4^ cells/well. They were then treated with EE or AE at different concentrations (100, 250, and 500 µg/mL) for 24 h. The medium was changed, and 100 µL of MTT at 1 mg/mL (dissolved in DMEM) was added. Cells were incubated for a further 4 h in a humidified 5% CO_2_ atmosphere at 37 °C. The liquid in the wells was aspirated off, and the formazan crystals were solubilized by adding 100 µL/well of ethanol. The absorbance at 570 nm was determined with a BioTek Epoch microplate spectrophotometer (Instruments Inc., Winooski, VT, USA). Cell viability was determined and compared to the negative control, which was cells incubated with only DMEM medium. This experiment was performed in triplicate and repeated three times. The percentage of cell viability was determined in relation to the negative control using the formula

% viability = (A _test_/A _control_) × 100

in which A _test_ is the absorbance of the experimental group, and A _control_ is the absorbance of the negative control.

### 4.6. Production of Intracellular ROS

The levels of intracellular ROS were evaluated by the quantification of the fluorescence emitted by 2′7′ dichlorofluorescein (DCFH-DA) [66].

The RAW 264.7 macrophage cells were grown in 96-well plates until they reached a density of 3 × 10^5^ cells/well with lipopolysaccharides (LPS) (2 μg/mL) for 18 h in a humidified 5% CO_2_ atmosphere at 37 °C. The medium was changed, and the EE and AE were added at different concentrations (100, 250, and 500 μg/mL), and they were incubated for a further 4 h in a humidified 5% CO_2_ atmosphere at 37 °C. The wells were washed with 1× PBS, and 100 μM DCFH-DA was added and incubated for another 1 h in a humidified 5% CO_2_ atmosphere at 37 °C in the dark. After this incubation, the fluorescence was read at 485 and 535 nm absorbance. The negative control (NC) cells were incubated with only DMEM, and positive control (PC) cells were incubated with LPS (2 µg/mL). This experiment was performed in triplicate and repeated three times. Results were expressed as % of fluorescence emitted in relation to negative control with LPS.

### 4.7. Dosage of Nitric Oxide

The immunomodulatory activity of *C. alnifolia* EE and AE was determined by quantifying the levels of nitrite released in the supernatant of RAW 264.7 macrophage cell cultures, as previously described [67]. Briefly, RAW 264.7 cells were grown at 3 × 10^5^ cells/well in 24-well plates. They were treated with EE and AE at 500 µg/mL for 24 h. Cells grown with LPS (2 µg/mL) were used as a positive control. After the treatment duration, 100 µL of the supernatant was collected and mixed with 100 µL of Griess reagent and incubated for 10 min at room temperature and protected from light. The absorbance was determined with a BioTek Epoch microplate spectrophotometer (Santa Clara, CA, USA) at 540 nm. The negative control (NC) cells were treated with only DMEM medium, and positive control (PC) cells were treated with only LPS (2 µg/mL). Sodium nitrite was used as a standard, and the results were expressed as a percentage of nitrite production in relation to the positive control (LPS), according to the formula

% nitrite production = (A _test_/A _LPS_) × 100

in which A _test_ is the absorbance of the experimental group, and A _LPS_ is the absorbance of LPS (positive control). This experiment was performed in triplicate and repeated three times.

### 4.8. Phagocytic Activity

The effect of EE and AE on the phagocytic activity was quantified as a complement to the immunomodulatory. This analysis was performed using the pHrodo ™ Red and Green BioParticles ^®^ Conjugates for Phagocytosis kit. The RAW 264.7 cells were grown in 96-well plates to approximately 2 × 10^6^ cells/well in a humidified 5% CO_2_ atmosphere at 37 °C. The EE and AE (500 µg/mL) were added to each well, and the plate was covered and kept in a humidified 5% CO_2_ atmosphere at 37 °C for 2 h to allow phagocytosis and acidification to reach their maximum. This experiment was performed in triplicate and read using a multilabel microplate reader GloMax^®^-Multi Detection System (Promega, Madison, WI, USA). The negative control (NC) cells were incubated with only DMEM medium, and positive control (PC) cells were incubated with LPS (2 µg/mL). The phagocytosis was calculated using the formula

Net phagocytosis was calculated: % Effect = _net experimental F_ × 100%/_positive control F liquid_

### 4.9. RNA Extraction and Quantitative PCR (qRT-PCR)

RAW 264.7 macrophage cell lines were grown in 25 cm^2^ bottles until they reached 2.5 × 10^6^ cells per bottle, then the EE and EA (500 µg/mL) were added, and it was maintained in a humidified 5% CO_2_ atmosphere at 37 °C for 24 h. Total RNA was then extracted using the ReliaPrep kit ^TM^ RNA cell Miniprep System (Promega) according to manufacturer’s instructions. The total RNA was quantified using a NanoDrop (Thermo Fisher Scientific—Waltham, MA, USA). The total RNA that had a ratio between 1.8 and 2.1 (A260/A280) was treated with DNAse (Ambion) according to the manufacturer’s instructions. The DNAse treatment was checked in accordance with Udvardi et al. [68]. Then, the cDNA was generated using 3 µg of total RNA and the High-Capacity cDNA Amplification kit from Applied Biosystems according to the manufacturer’s instructions. The qRT-PCR was completed using 5 μL 2× SYBR Green Master Mix, 15 ng of original cDNA, and 0.5 μM of each gene-specific primer (Table 3) in a 10 μL reaction using the QuantStudio5 from Applied Biosystems. Two constitutive genes were used for each plate (β-actin and elongation factor). Gene expression was normalized using β-actin gene. The RT-qPCR condition was 50 °C for 2 min, 95 °C for 10 min, 40 cycles of 95 °C for 15 s, and 60 °C for 1 min, followed by a melting curve. The experimental design involved three independent biological replicates and three technical replicates for each reaction. PCR efficiencies were calculated using LinRegPCR [69], threshold cycles (CT) were normalized for E = 2 [CT’ = CT × (log2 E/log2 2)], and errors bars correspond to the standard errors calculated as previously described [70]. The data obtained were analyzed using one-way ANOVA and Tukey’s test with *p* ≤ 0.05 in GraphPad Prism data analysis software version 5.0 (www.graphpad.com, accessed on 10 march 2020). The PCR efficiencies (E) were calculated using the *software* LinRegPCR 202110614, as previously described [69], and *threshold cycles* (Cts) were normalized to efficiency equal to 2 [Ct′ = Ct × (log _2_ E/log _2_ 2)]. Reverse and forward primers used for qRT-PCR are shown in Table 3.

### 4.10. Dosage of Cytokines

The experiment described in Section 4.7 was used for cytokine measurement. The supernatant from cells that were exposed to 500 µg/mL of EE or AE was used for the assay for cytokines quantification using BD kit Cytometric Bead Array (CBA) Mouse Th1/Th2/Th17 Cytokine Kit. In this assay, 50 µL from the supernatant was used, and it was quantified the presence of IL-17A and TNFα cytokines. This quantification was carried out according to the manufacturer’s instructions using the flow cytometer (FACSCanto II, BD Biosciences, San Jose, OR, USA).

### 4.11. In Vitro Scratch Assay

3T3 cell lines were grown in 24-well plates in DMEM with 10% FBS until a confluency of approximately 80–90% was obtained. The plates were kept in a humidified 5% CO_2_ atmosphere at 37 °C for 24 h. A linear scratch was created in a monolayer using a sterile 200 μL pipette tip. To remove cell debris, the wells were washed with phosphate-buffered saline (PBS, pH 7.4) and replaced with 200 µL of EE or AE at a final concentration of 500 µg/mL. Wells treated with only DMEM medium were used as a negative control (NC). Photographs were taken at 0, 8, 16, and 24 h after treatment with the extracts to observe the scratch closure. Images were obtained using a Nikon Eclipse inverted microscope with a 40× objective. The scratch area was analyzed using the NIS-Elements AR software ver5.42.01; the result is given as the percentage of scratch closure in relation to the initial area [71,72].

### 4.12. Antioxidant Assays

#### 4.12.1. Total Antioxidant Capacity (TAC)

This activity was measured based on the reduction of Mo^+6^ to Mo^+5^ by the AF and BF fractions. It was tested in three concentrations: 100, 250, and 500 μg/mL. The fractions were mixed with the 600 mM sulfuric acid ammonium molybdate. This mixture was kept at 100 °C for 90 min [73]. After that, the mixture was kept at room temperature to cool down, and the absorbance was read at 695 nm using the spectrophotometer BioTek Epoch Microplate (Santa Barbara, CA, USA). The results were compared to the negative control (distilled water). The total antioxidant capacity was expressed in equivalents of ascorbic acid (EAA/g). It was conducted in triplicate and repeated three times.

#### 4.12.2. DPPH

This assay measures the ability of the antioxidant molecules present in the samples to scavenge the radical DPPH [74]. It was tested the three concentrations of each fraction AF and BF (100, 250, and 500 μg/mL). The fraction was added at concentration tested to 100 μL of DPPH (0.1 mM). The sample and DPPH were mixed, and it was kept at room temperature for 30 min. After that, the samples were read using the spectrophotometer BioTek Epoch Microplate (Santa Barbara, CA, USA) at 517 nm. The blank control (B) was carried out with DPPH and ethanol 99.5% (Synth, Brazil), a control (C) was made only with DPPH, and the negative control was distilled water. The DPPH scavenging activity was calculated as follows: Scavenging Activity (%) = [(1 − (sample − blank))/negative control] × 100%. This assay was carried out in triplicate and repeated three times.

#### 4.12.3. Reducing Power

This assay is based on the reduction of potassium ferricyanide into potassium ferrocyanide by the samples evaluated [75]. The AF and BF fractions were tested in three concentrations: 100, 250, and 500 μg/mL. Sample was added to a solution having 0.2 M phosphate buffer (pH 6.6) and potassium ferricyanide (1% *w*/*v*) into a 4 mL final volume. The tube was kept at 50 °C for 20 min; then, the reaction was stopped by adding the TCA solution (10% *w*/*v*). The solution was then mixed with distilled water and ferric chloride (0.1% *w*/*v*). Phosphate buffer was used as blank control. The absorbance was measured at 700 nm using the spectrophotometer BioTek Epoch Microplate (Santa Barbara, CA, USA). The result was expressed as a percentage of the activity presented by 0.1 mg/mL ascorbic acid (Sigma-Aldrich, St. Louis, MO, USA). This assay was carried out in triplicate and repeated three times.

#### 4.12.4. Superoxide Scavenging Activity

The assay is based on the ability of the partitions phases (AF and BF) to inhibit the photochemical reduction of nitroblue tetrazolium (NBT) to riboflavinlight-NBT system [75,76]. The AF and BF fractions were evaluated at three concentrations: 100, 250, and 500 μg/mL. The fraction was added to 50 mM phosphate buffer (pH 7.8), 13 mM methionine, 100 mM EDTA, 75 mM NBT, and 2 mM riboflavin. After, the solution was exposed to a fluorescent lamp for 10 min. The change of color to blue was due to formazan production, and it was read in a spectrophotometer BioTek Epoch Microplate (Santa Barbara, CA, USA) at 560 nm. EDTA solution was used as a control, and distilled water was used as a blank. One of the two controls was made with an all-buffer reaction, and it was kept in the dark (B), and the second control (C) was made with an all-buffer reaction, and it was exposed to a fluorescent lamp for 10 min. Results were expressed as percent scavenging: Percentage of superoxide scavenging = {[(standard control − sample]/[standard control − blank]} × 100. This assay was carried out in triplicate and repeated three times.

#### 4.12.5. Copper Chelating

The AF and BF were evaluated in three concentrations: 100, 250, and 500 μg/mL. Fractions were mixed with 4 mM Pyrocatechol Violet in a sodium acetate buffer (pH 6.0) and copper sulfate pentahydrate (50 μg) [77]. This mixture was read at 632 nm using a spectrophotometer (Hitachi U-2000 Tokyo, Japan). Ultra-pure water was used as blank. This assay was carried out in triplicate and repeated three times.

### 4.13. Statistical Analysis

Statistical analysis was performed with Prism 6 software (GraphPad Prism version 6.00, Boston, MA, USA). Results were expressed as mean ± standard deviation (SD). Statistical differences between groups were evaluated using one-way ANOVA and Tukey’s test.

### 4.14. Partition Phase Analysis in HPLC Ms/Ms

The BF was resuspended into 5 mg/mL using methanol PA. The analysis was carried out using high-performance liquid chromatography with diode array detection and Ms/Ms (Shimadzu, Kyoto, Japan) coupled to a quadrupole mass spectrometer (Maxis) with a spray electron ionization (ESI) source operating in a positive mode. The column used was a Zorbax-C18 column (150 mm × 4.6 mm × 3.5 µm) at 40 °C. Solvent gradient was 0.1% acetic acid (AcOH) and acetonitrile (CH3CN), starting with 0% CH3CN for 0–5 min, then 0–10% for 5–12 min until reaching 10–20% for 12–22 min; then 20–100% for 22–35 min, while remaining at 100% for 5–40 min, ending with 8 min for post-run with 100% acetonitrile. The solvent flow rate was 1.0 mL × min^−1^, with a 5 µL injection volume. The ionization source conditions were ionization in positive mode, with capillary potential of 4000 V, sample drying temperature of 200 ^°^C, and nitrogen as a nebulizer gas flow of 5 L × min^−1^ with nebulizer gas pressure of 35 psi. Mass spectra were acquired in full scan mode in the range of *m*/*z* 50 to 1200. A collision energy ramp used for analysis was 20 to 75 eV at 352 and 280 nm.

### 4.15. Molecular Network

A molecular network was created using the online workflow (https://ccms-ucsd.github.io/GNPSDocumentation/ accessed on 21 September 2023) on the Global Natural Products Social Molecular Network (GNPS) website (http://gnps.ucsd.edu accessed on 21 September 2023). The data were filtered by removing all MS/MS fragment ions within +/− 17 Da of the precursor *m*/*z*. The MS/MS spectra were filtered by range, choosing only the top 6 fragment ions in the +/− 50 Da range across the spectrum. The precursor ion mass tolerance was set to 2.0 Da, and an MS/MS fragment ion tolerance of 0.5 Da.

### 4.16. Measurement of Intracellular ROS Induced by Oxidative Stress Generation and Image Analysis

This assay was done according to CEUA-279.006/2022 from Universidade Federal do Rio Grande do Norte (Brazil). The generation of ROS production in zebrafish was analyzed using the oxidation-sensitive fluorescent probe, DCFH-DA, according to reference [78], with modifications. Embryos, 8 h after fertilization, were transferred to 24-well plates in triplicate, containing 5 embryos per well (n = 15). These embryos were first treated for 1 h with the samples (EE, AE, AF, BF) at a concentration of 500 µg/mL and, after that, were treated with stressor agent peroxide of hydrogen (H_2_O_2_) for 24 h. The positive control (CP) was made up of embryos in nursery water and hydrogen peroxide, and the negative control (CN) was made up of embryos only with nursery water. After 24 h, the water in the wells was changed only to nursery water, and this plate was incubated for another 24 h. After this period, the DCFH-DA solution (20 µg/mL) was added to each well, and it was incubated for 1 h in the dark at 28.5 ± 1. After incubation, these animals (larvae) were anesthetized with 2-phenoxyethanol (Sigma dilution 1/500), and they were photographed under an inverted microscope. The fluorescence intensity of each larvae was quantified using the NIS-Elements software ver5.42.01 (Nikon Instruments, Cambridge, MA, USA).

## 5. Conclusions

The EE, AE, AF, and BF from *C. alnifolia* leaves were characterized by their antioxidant and immunomodulatory potential in this study. The data using the RAW 264.7 cell line showed that EE and AE were able to reduce ROS, NO, and phagocytosis, steps that are important in the inflammation process. In addition, the immunomodulatory activity by cytokines and mRNA expression was observed. The AF and BF were able to reduce NO. Furthermore, it was observed that EE, AE, AF, and BF were able to induce cell migration, especially at 24 h. In addition, these samples did not have toxicity in the zebrafish model and were protected against oxidative stress using an H_2_O_2_ stressor. However, the BF had an outstanding potential for closed wound healing compared to the four samples. In the future, other biological activities may be analyzed, and the bioactive molecules may be characterized to determine the potential of the *C. alnifolia* extracts and partition phases.

## Figures and Tables

**Table 1:** Phenolic compounds in the *C. alnifolia* partition phase suggested by HPLC-DAD.

Retention Time (min.)	Λ	Phenolic Compound by Library Comparison	AF	BF
2.000	280	galic acid	X	ND
10.257	280	*p*-cumaric acid	X	ND
13.576	280/352	vitexin	X	X

X—present; ND—not identified.

**Table 2:** Molecules annotated in BF from *C. alnifolia* by HPLC Ms/Ms.

t.r. (s)	Molecules	Mass [M+H]^+^	Mass Difference	Class
1218.59	Isoorientin	449.11	0	Flavonoid/Flavone
1440.46	2-(hydroxymethyl)-6-[4-(4-hydroxy-2,6,6-trimethylcyclohexen-1-yl)butan-2-yloxy]oxane-3,4,5-triol	375.24	0	
1334.56	Vitexin	433.11	0	Flavonoid/Flavone
2655.09	Haploperodide E	684.20	0.97	cumarin
2199.66	Kanakugiol	343.15	1.98	Chalcone
826.32	4-methylquinazoline-2-carboxamide	189.14	1.06	Alkaloid
2185.62	5-[(Z)-14-(3,5-dihydroxyphenyl)tetradec-8-enyl]benzene-1,3-diol	452.34	1.12	Polikedy
1497.61	tryptamine	243.19	1.98	Alkaloid

t.r. = retention time in seconds; mass [M+H]^+^” = correspond to molecular ion with the addition of a hydrogen; mass difference = difference in mass between the observed mass (HPLC/Ms-Ms) and the expected (GNPS) mass.

**Table 3:** Primers used for gene expression analysis.

Primer	Sequence
Elongation Factor (EF)	Reverse–5′ GGG TCA GAT TTC TTG AT′G GG 3′
	Forward–5′ CTG THE AG CGG CTG GCC′ AAG 3′
β-actin	Reverse–5′ CCT AGA AGC ATT TGC GGT GCA GAG 3′
	Forward–5′ TCA TGA AGT GTG CGT TGA CAT ′CG T 3′
Nitric Oxide Synthase (iNOS)	Reverse–5′ GTG CTT GTC ACC ACC AGC AGT AGT 3′
	Forward–5′ ACC TTG TTC AGC TCA GCC TTC AAC 3′
Interleukin 10 (IL-10)	Reverse–5′ TGG CAT GCT CAT TCA GCT CTT ATC 3′
	Forward–5′TAT GCA AGG ATT TGG TGT TGT TGG 3′;

## Data Availability

The data obtained in this study are available from the corresponding author upon request.

## Supplementary Materials

The supporting information can be downloaded at https://www.mdpi.com/article/10.3390/ijms242115885/s1.
