# Supplementary material for: The Antioxidant and Immunomodulatory Potential of Coccoloba alnifolia Leaf Extracts
Source: Int J Mol Sci. 2023 Nov 1;24(21):15885. doi: 10.3390/ijms242115885 (PMC10650087; doi:10.3390/ijms242115885)
Supplement: Supplementary file 1 [file ijms-24-15885-s001.zip › ijms-2679636-supplementary.pdf]

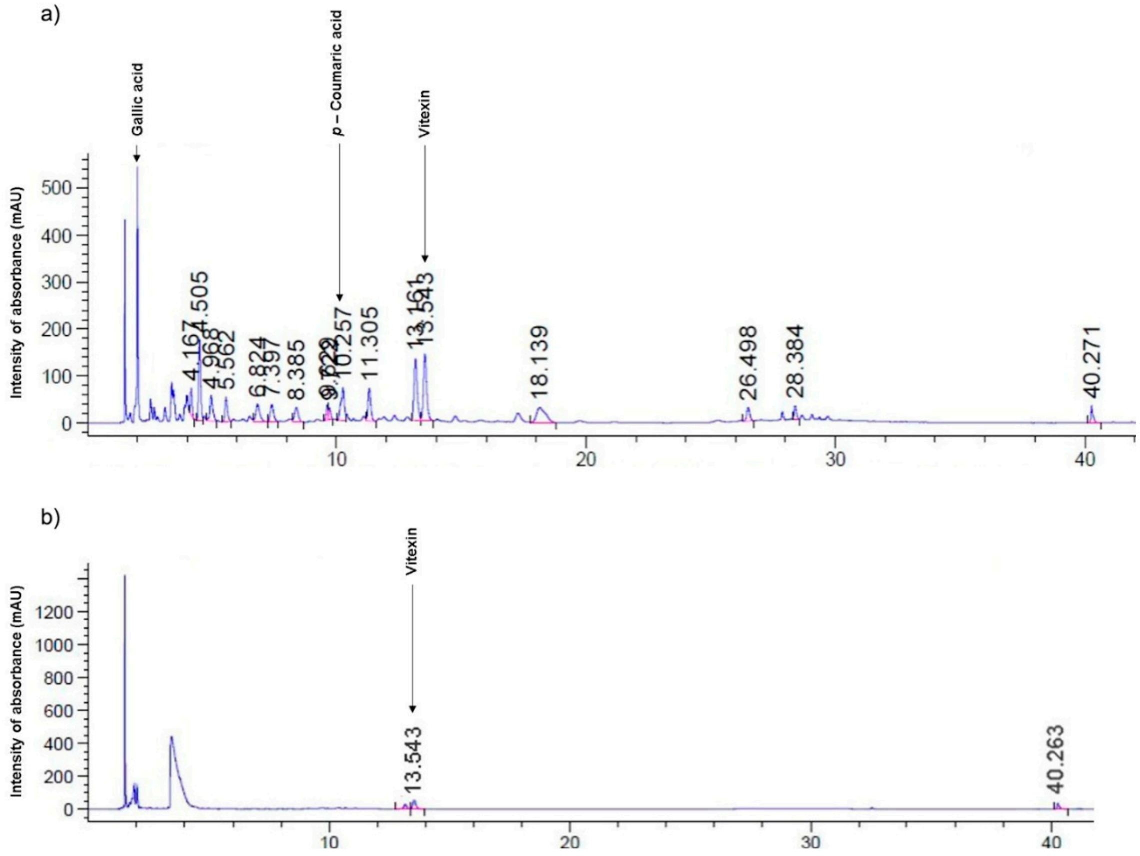

Supplementary Figure S1 Chromatogram of the substance profile of the FA and FB fractions of Coccoloba alnifolia in HPLC-DAD. In (a) FA chromatogram, showing the three substances in red, galic acid, p-coumaric acid and vitexin, respectively. In (B) FB chromatogram, showing the substance in red, vitexin.
